# Toxicological Analysis of Intoxications with Synthetic Cathinones

**DOI:** 10.1093/jat/bkab102

**Published:** 2021-10-01

**Authors:** Ewelina Pieprzyca, Rafał Skowronek, Piotr Czekaj

**Affiliations:** Department of Forensic Medicine and Forensic Toxicology, School of Medicine in Katowice, Medical University of Silesia, 18 Medyków Street, Katowice 40-752, Poland; Department of Forensic Medicine and Forensic Toxicology, School of Medicine in Katowice, Medical University of Silesia, 18 Medyków Street, Katowice 40-752, Poland; Department of Cytophysiology, Chair of Histology and Embryology, School of Medicine in Katowice, Medical University of Silesia, 18 Medyków Street, Katowice 40-752, Poland

## Abstract

Synthetic cathinones (SCs) are currently the second largest and the second most frequently seized group of new psychoactive substances. They are sold as replacements for controlled stimulants such as amphetamine, cocaine and 3,4-methylenedioxymethamphetamine. Administration of low doses of SCs can cause euphoria and increased alertness, and administration of high doses or chronic use of cathinones can cause serious adverse effects such as hallucinations, delirium, hyperthermia and tachycardia. In the years 2013–2019 in our practice, as many as 16 different SCs were detected in biological materials. This article lists the observed concentrations in 39 fatal and 18 non-fatal cases, in which a single SC as well as an SC in combination with amphetamine or ethyl alcohol were detected and quantified in biological materials. The quantitative analyses were carried out by liquid chromatography with tandem mass spectrometry. The analyzed cases of taking SCs were associated with intoxication (2 cases), fatal intoxication (36), driving under the influence of drugs (10) and other circumstances (9) such as violence, insulting an officer and holding a hostage. Taking SCs has serious side effects that can lead to multiple organ failure and death. Screening for the presence of SCs in biological materials should be part of the routine course of treatment in intoxication cases, both at the stage of clinical diagnosis and at the stage of forensic toxicological analysis. Ethyl alcohol and amphetamine may contribute to increased SC toxicity. These data could be valuable for further interpretation of other results from toxicological analyses.

## Introduction

Synthetic cathinones (SCs) are a group of psychostimulants commonly known as ‘bath salts’. This group derives from cathinone, which is one of the psychoactive ingredients of the plant called khat (*Catha edulis*). In terms of their chemical structure, SCs are derivatives of phenethylamine. They are one of the most commonly consumed stimulant and empathogenic substances, used as substitutes for amphetamine and 3,4-methylenedioxymethamphetamine (MDMA) as well as cocaine (1). Although only a few compounds from the cathinone group were known until recently, several years ago they began to dominate the illicit drug market. By 2018, there were 138 substances from the SC group reported to the EU Early Warning System (2). In recent years, the most popular SCs have been 4-methylmethcathinone (mephedrone, 4-MMC), 3,4-methylenedioxy-*N*-methylcathinone (methylone) and 4-methylenedioxypyrovalerone (MDPV). Mephedrone has been the most common SC in Europe, while MDPV and methylone have been most common in the United States.

On the illicit market, SCs are usually distributed in the forms of white powders, crystals, capsules or tablets. They are usually taken orally and nasally, while intravenous and intramuscular injections and rectal infusions are less popular. They can be usually purchased in the form of white, beige or brown powdered substance, placed in a small resealable bag and described as ‘not for human consumption’, ‘jewelry cleaner’, ‘plant food’ or ‘phone screen cleaner’. Newer solutions are also available, namely ready-to-use nasal sprays and electronic cigarette liquids. The main distribution channels for these substances are now online shops, where they can be purchased under various names, including ‘Flakka’, ‘Vanilla Sky’, ‘Cherry Cocolino’, ‘Ivory Wave’ or so-called ‘research chemicals’ (3, 4).

SCs represent a broad class of pharmacologically active compounds that produce a variety of effects through different mechanisms of action. Therefore, each case of intoxication with an SC should be assessed individually, and each subsequently described intoxication case provides invaluable information, such as about the identified concentration. Administration of low doses of SCs can cause euphoria and increased alertness, and administration of high doses or chronic use of cathinones can cause serious adverse effects such as hallucinations, delirium, hyperthermia and tachycardia. Repeated use of SCs can result in paranoia and hallucinations, and in some patients, it can lead to the so-called ‘excited delirium’, a syndrome with signs and symptoms of extreme psychomotor agitation and aggressive behavior toward self and the environment (5). Other signs and symptoms of intoxication include dehydration, muscle damage and renal failure, which can result in multi-organ failure and death (6).

The growing numbers of SCs on the illicit drug market and of their users pose a challenge for forensic and clinical toxicologists but also for legislators. While new compounds keep appearing on the drug market, information about their potential toxicity is scarce and the number of drug emergencies associated with their use is rising. SC derivatives can be much more potent than classic drugs and their ingestion can cause sudden death (7). Acute and chronic toxicity of many SCs is still poorly understood or even completely unknown, and research in this area is relatively scant. Given the increasingly complex threat to public health, interdisciplinary and coordinated action is needed to explain acute and chronic effects of synthetic drug use.

This article describes the results of analyses of 57 cases from own practice, in which a single SC as well as an SC in combination with amphetamine or ethyl alcohol were detected and quantified in blood. Cases of simple intoxication are particularly useful for assessing health effects of SCs because they eliminate the consideration of possible interactions between two or more simultaneously used xenobiotics. In a small number of cases, drugs were detected. This work may help to interpret specific concentrations of SCs and determine the effects of these substances, which may have practical applications.

## Experimental

### Materials and methods

A toxicological analysis was performed of 5,001 cases, including 868 women and 4133 men (involving mainly blood and urine samples). From these cases, 57 cases were selected in which SCs were detected and determined in blood and other biological materials. These cases involved use of a single SC as well as an SC in combination with amphetamine or ethyl alcohol.

### Certified reference materials and reagents

Certified reference materials were purchased from Cayman Chemical Company (Ann Arbor, MI, USA), Cerilliant (Round Rock, TX, USA), LGC Standards (Dziekanów Leśny, Poland) and Lipomed (Arlesheim, Switzerland).

The remaining reagents used in the analyses were of sufficient purity (high-performance liquid chromatography grade).

### Biological materials—samples

The subject of the study were biological materials (including blood, urine and vitreous humor samples as well as liver and kidney tissues) delivered to the Department of Forensic Medicine and Toxicology of the Medical University of Silesia in Katowice or collected locally during medico-legal autopsies in the years 2013–2019. In each case, the abovementioned department received the decision of the police or the prosecutor’s office to perform toxicological analysis for the presence of psychoactive substances. Most of the analyzed cases concerned intoxication, including fatal intoxication, suicide, driving under the influence of psychoactive substances and possession of drugs. The obtained results of toxicological analyses were compared with the data from protocols of biological material collection and data from prosecutor’s and court files of completed cases. The case files were each time made available for analysis with the consent of the competent prosecutor’s office or court.

### Analytical methods

Routine analyses for the common drugs of abuse and prescription drugs involved the use of enzyme-linked immunosorbent assay, high-performance liquid chromatography coupled with diode array detection and liquid chromatography with mass spectrometry. New psychoactive substances (NPS), including SCs, were identified and quantified by liquid chromatography coupled with a triple quadrupole mass spectrometer—Thermo Scientific TSQ Quantum Access MAX. The analytes were separated in a Thermo Scientific C18 column (150 × 2.1 mm ID, 5 μm). The mobile phase was fed into the column in gradient mode in the form of a mixture of 0.2% formic acid solution in water (v/v) and 0.2% formic acid solution in acetonitrile (v/v). The mass spectrometer was operated in the positive ion detection mode using full scan acquisition and multiple reaction monitoring. The analytes were isolated from the matrix by acetonitrile precipitation as well as extraction with ethyl acetate. The exact test procedure was published together with the detailed description of two cases of intoxication with the PV8 SC (8).

## Results

The vast majority of SC users were men (88%, 50 cases), with women accounting for only 12% (7 cases). Considering the total number of cases analyzed from 2013 to 2019, 17.4% of all cases were females. User age ranged from 18 to 52 years, with a mean age of 31.1 years and a median age of 30.0 years. In several cases, the age of users was unknown.

The analyzed cases of taking SCs were associated with intoxication (*n* = 2), fatal intoxication (*n* = 36), driving under the influence of drugs (DUIDs) (*n* = 10) and other circumstances (*n* = 9) such as violence, insulting an officer and holding a hostage.

In the analyzed cases, the following substances were identified (number of determinations in brackets): α-pyrrolidinopentiophenone (α-PVP) (*n* = 13), 3-methylmethcathinone (3-MMC) (*n* = 10), 4-chloromethcathinone (4-CMC) (*n* = 10), 4-methyl-α-ethylaminopentiophenone (4-MEAP) (*n* = 6), 4-chloroethcathinone (4-CEC) (*n* = 2), pentedrone (*n* = 3), PV8 (*n* = 2), *N*-ethylbuphedrone (NEB) (*n* = 2), *N*-ethylcathinone (*n* = 2), α-pyrrolidinopentiothiophenone (α-PVT) (*n* = 1), *N*-ethylpentylone (*n* = 1), 4-methylenedioxy-α-pyrrolidinohexanophenone (3,4-MDPHP) (*n* = 1), 4-methylethcathinone (4-MEC) (*n* = 1), α-ethylaminopentiophenone (*N*-ethylpentedrone) (*n* = 1), 4-MMC (mephedrone) (*n* = 1) and 3,4-dimethylmethcathinone (3,4-DMMC) (*n* = 1). In the years 2013–2014, the following substances were determined in samples of biological material: 3,4-DMMC, 4-MEC, 4-MMC, pentedrone, 3-MMC, NEB, α-PVP and N-ethylcathinone. In the following two years, α-PVP and 3-MMC were identified most frequently, followed by the abovementioned PV8, α-PVT, *N*-ethylpentedrone and 4-CMC. In the years 2017–2019, in addition to 4-CMC and *N*-ethylpentedrone, testing revealed the presence of 4-MEAP, 3,4-MDPHP, 4-CEC and *N*-ethylpentylone.

**Figure 1.**
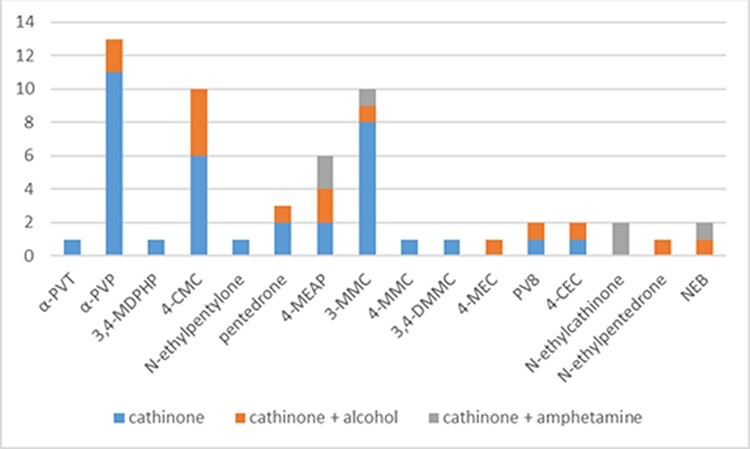
Cases of detection of synthetic cathinones used alone or in combination with amphetamine and/or ethyl alcohol (*n* = 57).

Figure 1 shows the different types of identified SCs and distinguishes between an SC used alone or in combination with amphetamine and/or alcohol. Most of the analyzed cases concerned isolated use of one type of SC (simple intoxication accounted for over 63%). More than 26% of the analyzed cases involved the use of an SC with ethyl alcohol, and slightly more than 10% of the cases concerned the use of an SC with amphetamine.

The trade names of the products taken by the users were obtained only in a few cases. In the biological materials of the man who supposedly used the ‘M3’ product, testing revealed the presence of α-PVP; the ‘MCH’ product was identified as 3-MMC, and products labeled as ‘Product Imitation: Red Dirt’ and ‘Ruby Sand Additive, 0.5 Gram—Product Imitation’ were identified as 3,4-DMMC and 3-MMC, respectively.

On the Polish illicit drug market, ‘crystal’ is synonymous with virtually any substance. In the biological materials of users who reported taking ‘crystal’, testing revealed the presence of *N*-ethylpentylone, pentedrone, α-PVP, 3-MMC and 4-CEC.

In one of the analyzed cases, the man reported taking amphetamine, while examination of his blood revealed the presence of α-PVP.

The determined blood concentration ranges of SCs are shown in Table I.

**Table I.** Synthetic Cathinone (SC) Concentrations for Blood Samples in Cases of an SC Used Alone or in Combination with Amphetamine or Ethyl Alcohol (*n* = 57)

Substance	Concentration range (ng/mL)	Mean concentration (ng/mL)	Median concentration (ng/mL)
α-PVT	410	–	–
α-PVP	20–1,049	284	130
3,4-MDPHP	390	–	–
4-CMC	6–242	93	34
*N*-ethylpentylone	1,320	–	–
Pentedrone	142–817	450	392
4-MEAP	72–3,121	1,610	1,622
3-MMC	12–5,310	1,212	328
4-MMC	8,404	–	–
3,4-DMMC	5,100	–	–
4-MEC	202	–	–
PV8	70–260	165	165
4-CEC	210–1,633	922	922
*N*-ethylcathinone	519–3,504	2,012	2,012
*N*-ethylpentedrone	932	–	–
NEB	53–212	133	133

Almost 70%, i.e., 39 of the analyzed cases, were fatal. Such a high mortality rate in the analyzed group may be related to the types of biological materials provided for toxicological testing—mostly materials collected during autopsy. The analyzed cases of SC use with the determined concentrations are listed in Tables I and II.

**Table II.** Synthetic Cathinone Concentrations for Blood Samples—Fatal (*n* = 39) and Non-fatal Cases (*n* = 18)

Substance	Fatal cases (*n* = 39)				Non-fatal cases (*n* = 18)			
	Number of determinations	Concentration range (ng/mL)	Mean concentration (ng/mL)	Median concentration (ng/mL)	Number of determinations	Concentration range (ng/mL)	Mean concentration (ng/mL)	Median concentration (ng/mL)
α-PVT	1	410	–	–	–	–	–	–
α-PVP	7	20–544	184	62	6	44–1,049	401	186
3,4-MDPHP	1	390	–	–	–	–	–	–
4-CMC	8	6–242	112	94	2	13–23	18	18
*N*-ethylpentylone	1	1,320	–	–	–	–	–	–
Pentedrone	2	392–817	605	605	1	142	–	–
4-MEAP	4	72–3,121	1,610	1,622	1	390	–	–
3-MMC	4	391–5,310	2,691	2,531	6	12–344	226	267
4-MMC	1	8,404	–	–	–	–	–	–
3,4-DMMC	1	5,100	–	–	–	–	–	–
4-MEC	–	–	–	–	1	202	–	–
PV8	2	70–260	165	165	–	–	–	–
4-CEC	2	210–1,633	922	922	–	–	–	–
*N*-ethylcathinone	1	3,504	–	–	1	519	–	–
*N*-ethylpentedrone	1	932	–	–	–	–	–	–
NEB	2	53–212	133	133	–	–	–	–

### Cases of non-fatal SC use

Supplementary Table S1 presented in the Supplementary Material describes cases of non-fatal use of SCs along with their concentrations.

The cases in Supplementary Table S1 concern DUIDs, intoxication, attempted suicide and other crimes.

In the analyzed DUID cases, four types of SCs were determined: α-PVP, 3-MMC, 4-CMC and 4-MEAP. In drivers who were under the influence of α-PVP and 4-CMC, these substances were determined in concentrations of 44–1,049 and 13 ng/mL, respectively; 3-MMC was revealed in the concentration range of 12–344 ng/mL, and 4-MEAP was found in the concentration of 390 ng/mL.

Not all protocols of blood collection from drivers were filled in, and in some cases, no abnormal signs or symptoms were noted, which may be related, for example, to the development of tolerance to a given substance. For case no. 10, it was noted in the blood collection protocol that the patient had dilated pupils with poor light reflex. Pupil dilation was also noted in a patient who had a suicide attempt after taking pentedrone and ethyl alcohol (case no. 16) and in case no. 1 after taking 4-MEC and ethyl alcohol.

Case nos. 5 and 17 concerned intoxication with, respectively, α-PVP and *N*-ethylcathinone. The measured blood concentration of α-PVP was 890 ng/mL. The noted signs and symptoms of the action of this substance were as follows: psychomotor agitation, elevated heart rate and involuntary movements. The man was unable to walk unassisted—he was strongly affected by α-PVP. In addition to α-PVP, testing revealed the presence of midazolam at a concentration of 10 ng/mL, which could have been administered during medical assistance. *N*-ethylcathinone determined at a concentration of 519 ng/mL caused elevated heart rate and depressed mood. In addition to *N*-ethylcathinone, testing identified amphetamine at a concentration of 90 ng/mL. The baseline concentration of *N*-ethylcathinone and amphetamine may have been higher because blood was collected for analysis only 5 hours after consuming the drug.

The remaining cases in Supplementary Table S1 were related to criminal activities committed after taking 4-MEC, α-PVP or 3-MMC.

### Fatal cases excluding suicides

Supplementary Table S2 presented in the Supplementary Material shows cases of fatal intoxication and road traffic fatalities.

In the analyzed 39 cases of fatal intoxication and road traffic fatalities, the following substances were determined in the autopsy materials: 4-CMC (*n* = 8), α-PVP (*n* = 7), 4-MEAP (*n* = 5), 3-MMC (*n* = 4), 4-CEC (*n* = 2), pentedrone (*n* = 2), PV8 (*n* = 2), NEB (*n* = 2), *N*-ethylpentedrone (*n* = 1), *N*-ethylpentylone (*n* = 1), 3,4-DMMC (*n* = 1), 4-MMC (*n* = 1), 3,4-MDPHP (*n* = 1), α-PVT (*n* = 1) and *N*-ethylcathinone (*n* = 1). The co-presence of ethyl of alcohol in the blood was found in 13 cases (the maximum concentration was 3.2 mg/mL). In six cases, the presence of amphetamine was determined (combined with 4-MEAP, *N*-ethylcathinone, NEB, 3-MMC and α-PVP), of which two cases showed it only in urine samples. The deaths concerned people aged 18–52 years. In most cases, the victims were drug addicts.

The largest number of cases concerned fatal intoxication after consuming 4-CMC and α-PVP. The presence of 4-CMC was determined in postmortem blood in the concentration range of 6–242 ng/mL, with the range of 6–223 ng/mL representing the subgroup of fatal intoxication incidents. In cases of fatal intoxication caused by the toxic effects of α-PVP, this compound was determined in postmortem blood in the concentration range of 20–544 ng/mL.

In the reported four cases of fatal intoxication after taking 3-MMC, the concentration determined in postmortem blood ranged from 391 to 5,310 ng/mL. In case no. 2, blood concentration was high (5,310 ng/mL), while urine concentration was almost four times lower, which may confirm the information in the records that the drug was taken intravenously shortly before death. It is also worth noting case no. 3, concerning a woman who supposedly took about 400 mg of 3-MMC dissolved in water. The postmortem concentration of 3-MMC was determined at 3,352 ng/mL in blood and at 748 ng/mL in vitreous humor. The woman supposedly took 3-MMC for the first time, and her death was preceded by convulsions.

## Discussion

The study discusses 57 cases where SCs were detected and determined in blood and other biological materials. These cases concerned the use of a single SC (simple intoxication) as well as the use of an SC together with amphetamine or ethyl alcohol. User age ranged from 18 to 52 years, with a mean age of 31.1 years and a median age of 30.0 years. Similar data regarding the age of SC users were also reported by other authors (9–11).

In the years 2013–2019, as many as 16 different SCs were detected in biological materials. The order of appearance of the next SCs was similar to that presented by Adamowicz et al. (12) and was mainly related to the introduction of new additions to the list of controlled substances and the imposition of legal sanctions in Poland, Europe and globally.

Only in a few cases information was obtained regarding the route of SC intake (nasal, oral, injections, water solution, crystal wrapped in paper—so-called ‘bombing’); the identified intake routes were consistent with data from the literature (3). The cases presented by us confirm the assumption that users of psychoactive substances do not always know what products they are taking. Unintentional use of an SC significantly increases the risk of overdose or even death. The literature has already reported cases of fatalities due to overdose of SCs following unintended use of ‘bath salts’ (13).

The collected cases were divided into two groups—non-fatal (survivable) and fatal cases. The former group was dominated by drivers. Driving under the influence of SCs is becoming increasingly common, which is a particularly relevant problem with regard to road safety. Users reaching for NPS expect similar effects to those produced by conventional drugs, often forgetting about the side effects of these substances. The use of SCs may lead to psychomotor disturbances similar to those caused by amphetamine or MDMA, and driving after their intake can affect safe driving and poses a high risk to the driver, passengers and other road users (14). For ethical reasons, it is unlikely that driving simulation tests will be conducted after administering specific NPS to volunteers; therefore, knowledge about possible psychomotor disturbances should be drawn by analyzing real examples. These observations are difficult, not carried out under controlled conditions and influenced by many factors. In addition, the number of cases is limited, the tolerance of subjects is unknown, the doses of ingested substances and the time of ingestion are unknown; usually more than one substance is present in the biological material.

The data presented in the literature show that drivers who were driving under the influence of substances from the SC group exhibited, among others, disorientation, aggression, agitation, tachycardia, walking difficulties, dizziness, speech problems, facial skin redness and dilated or narrow pupils with poor light reflex (15, 16).

The data presented by us come from real road traffic incidents, where drivers were most often stopped due to suspicious driving. Most of the cases concerned driving under the influence of SCs alone, with the exception of case 15 in Supplementary Table S1, where ethyl alcohol was also identified.

The concentrations determined in cases of driving, causing a road accident, or possession of drugs can be regarded as concentrations that are typical for cases of intoxication with these substances. Other non-fatal incidents and intoxication cases can also be included in the same group. The concentration range determined for DUIDs and α-PVP was wide, and the value determined for case no. 7 was high compared to the data available in the literature (11, 17, 18). The upper limit of the concentration range determined for 3-MMC (344 ng/mL) was higher than in the cases of materials collected from drivers and analyzed by Adamowicz et al., where 3-MMC was determined in concentrations ranging from 1 to 171 ng/mL (19). The identified concentration of 4-CMC (13 ng/mL) was within the concentration range (1.3–75.3 ng/mL) described in the DUID cases described by Tomczak et al. (20). In case no. 18, 4-MEAP was determined at a concentration of 390 ng/mL, and the driver showed signs of drowsiness, irritability and poor pupillary light reflex.

In most non-fatal cases, SC concentrations were as high as hundreds of nanograms per milliliter. The higher concentrations observed in living people may be caused by SC addiction and the development of tolerance to a given substance or may be due to blood sampling shortly after SC administration. When interpreting high SC concentrations, it is also important to consider cases of hospitalized patients who survived, thanks to receiving adequate intensive medical care (e.g., case no. 5 in Supplementary Table S1).

Supplementary Table S2 lists 39 cases of fatal intoxication and road traffic fatalities following SC use. The co-presence of ethyl alcohol in the blood was found in 13 cases (the maximum concentration was 3.2 mg/mL). In six cases, the presence of amphetamine was determined (combined with 4-MEAP, *N*-ethylcathinone, NEB, 3-MMC and α-PVP), of which two cases showed it only in urine samples.

The signs and symptoms that preceded SC-related deaths included agitation, nervousness, aggression, excessive sweating, shivering, slurred speech, vomiting, blood leakage from the nose and mouth, loss of consciousness, hallucinations, tightening of the body and pupil dilation.

In two cases of death after taking α-PVP (case nos. 27 and 31), police officers intervened due to irrational and aggressive behavior. Similar cases were also noted after taking *N*-ethylpentylone (case no. 33) and 4-MEAP and amphetamine (case no. 16). In the four cases mentioned above, there is suspicion of excited delirium syndrome, whose typical signs and symptoms are, among others, strange and aggressive behavior, screaming, paranoia, panic, violence against others, unexpected and above-average physical strength, and hyperthermia (21).

The largest number of cases concerned fatal intoxication after taking 4-CMC and α-PVP. The presence of 4-CMC was determined in postmortem blood in the concentration range of 6–242 ng/mL, with the range of 6–223 ng/mL representing the subgroup of fatal intoxication incidents. In the fatal cases, the determined concentration range for 4-CMC was lower than that shown by Tomczak et al. (56.2–1,870 ng/mL) (20).

In the cases of fatal intoxication caused by the toxic effects of α-PVP, this compound was determined in postmortem blood in the concentration range of 20–544 ng/mL. Similar concentrations were reported by Potocka et al. (174 ng/mL) (22) and Nagai et al. (411 ng/mL) (23). In some studies, the concentration range found in fatal intoxication was higher; for example, Adamowicz et al. reported fatal cases of α-PVP use for concentrations of 1.1–6,200 ng/mL (11).

In addition to the cases of deaths occurring after taking most popular SCs, such as mephedrone, 3-MMC, pentedrone, α-PVP and 4-CMC, which have already been described in the literature, this study also includes less popular SCs. To date, the literature has described very few or no cases of death following the use of NEB, *N*-ethylcathinone, α-PVT, 3,4-MDPHP, *N*-ethylpentylone or 3,4-DMMC.

As can be easily seen in Table II, the concentration ranges in cases of non-fatal and fatal SC use may overlap. This is an extremely important observation for consultative practice. Cases of 3-MMC, 4-CMC and α-PVP use included deaths where these substances were found in very low concentrations. There could have been several reasons for this. Death may have occurred after a longer period and intensive life-saving procedures, which possibly lowered the blood concentration of the xenobiotic. Low SC levels in fatal cases may also be due to other reasons, such as the presence of additional substances and interactions between them, individual variability, intolerance to a given substance or the presence of additional diseases. It should also be borne in mind that the toxic effect may also occur at low concentrations. However, the mean and median concentrations in the fatalities were significantly higher for 3-MMC and 4-CMC than for the other types of incidents. No such relationship was observed for α-PVP due to the high concentration determined in the driver (case no. 7 in Supplementary Table S1).

The correct interpretation of the analytical result depends on many components that must considered by an expert. Analyses of SC concentrations should take into account the stability of these substances in biological materials. Literature data indicate that many SCs are unstable in biological materials (24). The final SC concentration may, therefore, be lower compared to that at the time of death or sampling of analytical materials. In addition, the results of xenobiotic concentrations in autopsy materials should be interpreted carefully, taking into account the circumstances of death (e.g., resuscitation), substance metabolism, and place and time of sampling for analysis (possibility of postmortem redistribution).

The cases analyzed in this study concerned the use of a single SC as well as the use of an SC in combination with amphetamine or ethyl alcohol. About 26% of the cases analyzed in this article concerned a combination of an SC and ethyl alcohol. Drug users often combine them with alcohol despite having little knowledge of the substance they are taking. Papaseit et al. described the interaction of ethyl alcohol and mephedrone in humans. They found out that the combination of mephedrone and alcohol increased the cardiovascular effects of mephedrone and produced a more intense feeling of euphoria and well-being compared to isolated use these substances. Mephedrone reduced the sedative effects of alcohol (25). McGaw et al. indicated in their study that mephedrone in combination with alcohol may increase the risk of arrhythmias (26). O’Neill et al. conducted socio-epidemiological studies on the concurrent use of mephedrone and ethyl alcohol. Most of the study participants declared that they consumed high doses of alcohol prior to taking mephedrone and then reduced alcohol intake. In addition, some study participants believed that mephedrone cancelled out the effects of alcohol, and the ability to drink alcohol without experiencing its full effects was seen as beneficial. Some respondents believed that alcohol-induced effects gave them courage to take mephedrone (27).

In the analyzed cases of SC that ended with death and where the co-presence of ethyl alcohol was demonstrated (*n* = 13), its concentration was determined in the range of 0.3–3.2 mg/mL, that is, in the range below lethal concentrations (above 3.5 mg/mL) (28).

Another subgroup of cases were the deaths after taking an SC and amphetamine. In the analyzed cases of SC use that ended with death and where the co-presence of amphetamine was demonstrated (*n* = 6), its concentration was determined in the range of 50–400 ng/mL covering normal (20–100 ng/mL) and toxic concentrations (200 ng/mL). The lethal concentration range for amphetamine was determined above 500 ng/mL [28]. The co-presence of amphetamine was reported in cases of use of 3-MMC, 4-MEAP, NEB, *N*-ethylcathinone and α-PVP. In three cases, information was obtained that these persons were long-time drug addicts (case nos. 2 15, and 16 in Supplementary Table S2). In case nos. 2, 16, 23 and 30, high SC concentrations were reported.

The literature describes the effect of co-administration of amphetamine and mephedrone in mice (29). Over the course of 8 days, the mice received seven injections of a mixture of amphetamine at a dose of 1.0 mg/kg and mephedrone at a dose of 3.0 mg/kg body weight; in two separate groups, they received injections of amphetamine at a dose of 1.0 mg/kg or mephedrone at a dose of 3.0 mg/kg body weight. Then, after a 10-day interval, the mice were treated in a similar manner with amphetamine at a dose of 1.0 mg/kg body weight. It was shown that repeated co-exposure to amphetamine and mephedrone produced a stronger sensitization reaction compared to repeated exposure to each substance administered separately. In addition, sensitization to successive provoking doses of amphetamine after a 10-day interval was stronger in the rodent group pretreated with mephedrone or a mixture of mephedrone and amphetamine and weaker in rodents pretreated with amphetamine. The obtained results can be interpreted as suggesting that mephedrone and amphetamine have additive effects when administered concurrently. In addition, preliminary findings suggest that the abuse potential of mephedrone may be increased when combined with other psychostimulants.

Considering the abovementioned information presented by Berquist et al. and the high SC concentrations obtained in four actual cases, it can be concluded that the potential for SC abuse may be increased when combined with amphetamine (29).

Determination of fatal intoxication requires quantification of potentially lethal substances and exclusion of alternative causes of death. The results of postmortem toxicological analyses should always be interpreted individually in the context of all information on the case.

There are no characteristic autopsy findings in people intoxicated with legal highs. Most often, the observations are limited to markers of sudden death with features of acute circulatory failure in the form of brain and lung edema, internal organ congestion and blood fluidity. The scope of the encountered lesions depends mainly on the age of the deceased and whether the intoxicated person was hospitalized.

## Conclusion

The obtained data on the types of SCs detected in biological materials reflect the most popular NPS identified in ‘legal highs’ in Poland and Europe during the analyzed period.

In the drivers who were driving under the influence of SCs, the reported side effects included confusion, drowsiness and irritability. SCs can increase the propensity to make risky decisions as well as affect concentration and cause visual disturbances.

Based on the analysis of the results of toxicological tests and analysis of case files, 39 cases were identified of deaths related to isolated use SCs or their combination with ethyl alcohol or amphetamine. Taking SCs has serious side effects that can lead to multiple organ failure and death. Screening for the presence of SCs in biological materials should be part of the routine course of treatment in intoxication cases, both at the stage of clinical diagnosis and at the stage of forensic toxicological analysis. Ethyl alcohol and amphetamine may contribute to increased SC toxicity.

## Supplementary Material

bkab102_Supp
