# Supplementary material for: Toxicological Analysis of Intoxications with Synthetic Cathinones
Source: J Anal Toxicol. 2021 Oct 1;46(7):705–11. doi: 10.1093/jat/bkab102 (PMC9375235; doi:10.1093/jat/bkab102)
Supplement: bkab102_Supp [file bkab102_supp.zip › jat-21-3492-File002.doc]

Table S1. Cases of non-fatal synthetic cathinone use

| Case number | Substance and blood concentration range [ng/mL] | Sex/age | Case description | Other drugs (blood concentration in ng/mL, alcohol in mg/mL) |
| --- | --- | --- | --- | --- |
| 1 | 4-MEC: 202 | M/U | The man insulted a police officer.  The signs and symptoms noted in the blood collection protocol: rowdy mood, dilated pupils, and normal pupillary light reflex. | alcohol: 1.45 |
| 2 | α-PVP: 130 | F/U | The woman was pushed out of the window by a friend. The signs and symptoms noted in the blood collection protocol: psychomotor agitation, slurred speech, unable to walk |  |
| 3 | α-PVP: 241 | M/U | He pushed a female acquaintance from a flat balcony. Before the incident, he had been taking drugs. He said he had taken amphetamine.  The signs and symptoms noted in the blood collection protocol: cheerful mood, normal pupils, and normal pupillary light reflex. |  |
| 4 | α-PVP: 44 | M/U | Driver. |  |
| 5 | α-PVP: 890 | M/33 | Intoxication. The man used the ‘M3’ product intravenously.  The signs and symptoms noted in the blood collection protocol: psychomotor agitation, elevated heart rate, unable to walk, and involuntary movements. | midazolam: 10 |
| 6 | α-PVP: 52 | M/18 | The man caused a false bomb alarm. He had been using ‘crystal’. |  |
| 7 | α-PVP: 1,049 | M/37 | Driver - the man was stopped for roadside check.  The man suffered from paranoid schizophrenia. | amisulpride: 7 |
| 8 | 3-MMC: 311 | M/41 | The man was holding a hostage. |  |
| 9 | 3-MMC: 12 | M/U | Driver - the man was stopped for roadside check. He took the ‘MCH’ powder. |  |
| 10 | 3-MMC: 293 | M/20 | Driver.  The signs and symptoms noted in the blood collection protocol: dilated pupils and weak pupillary light reflex. |  |
| 11 | 3-MMC: 240 | M/29 | Driver.  The signs and symptoms noted in the blood collection protocol: regular pulse, normal pupils, and normal pupillary light reflex. |  |
| 12 | 3-MMC: 344 | M/25 | Driver.  The signs and symptoms noted in the blood collection protocol: regular pulse and rowdy mood. |  |
| 13 | 3-MMC: 155 | M/34 | Driver.  The signs and symptoms noted in the blood collection protocol: regular pulse, normal pupils, normal pupillary light reflex, positive Romberg test, and positive finger–nose test. |  |
| 14 | 4-CMC: 23 | F/19 | Passenger of the car. The woman took one line of white powder intranasally three hours before blood sampling for analysis. |  |
| 15 | 4-CMC: 13 | M/21 | Driver - road accident participant. | alcohol: 0.96 |
| 16 | pentedrone: 142 | M/26 | Suicide attempt—hanging.  The signs and symptoms noted in the blood collection protocol: regular pulse and dilated pupils. | midazolam: 50; lidocaine: 140; alcohol: 2.0 |
| 17 | N-ethylcathinone: 519 | M/U | Intoxication. The man took ‘crystal’. Blood was drawn five hours after drug use.  The signs and symptoms noted in the blood collection protocol: depressed mood, elevated heart rate, normal pupils, and normal pupillary light reflex. | amphetamine: 90 |
| 18 | 4-MEAP: 390 | M/25 | Driver.  The man was stopped for roadside check—drowsy, irritable, poor pupillary light reflex. |  |

U – unknown, M – men, F – female
