# Supplementary material for: Toxicological Analysis of Intoxications with Synthetic Cathinones
Source: J Anal Toxicol. 2021 Oct 1;46(7):705–11. doi: 10.1093/jat/bkab102 (PMC9375235; doi:10.1093/jat/bkab102)
Supplement: bkab102_Supp [file bkab102_supp.zip › jat-21-3492-File003.doc]

Table S2. Cases of fatal synthetic cathinone use

| Case number | Substance and blood/urine/vitreous humour concentration range [ng/mL] | Sex/age | Case description | Other drugs (blood/urine/ vitreous humour concentration in ng/mL, alcohol in mg/mL) | Cause of death/PMI |
| --- | --- | --- | --- | --- | --- |
| 1 | 3-MMC: 391 B, 64 Ur | F/34 | The body was found in a flat.  The woman came to her friends’ flat under the influence of drugs, she was agitated, shivering, nervous, and her speech was slurred. She was addicted to alcohol and drugs. | alcohol: 0.3 B, 0.6 Ur | acute circulatory and respiratory failure in the course of intoxication/5 days |
| 2 | 3-MMC: 5,310 B, 1,361 Ur | M/U | The body was found in the basement.  At the scene of the discovery, there were needles, a syringe with a depressed plunger, small plastic bags labelled ‘ruby sand additive 0.5 gram—product imitation’, small empty resealable bags with traces of colourless liquid, and empty mineral water bottles. The man had a history of using drugs: legal highs, amphetamine, and cannabis, which he used in the form of smoking mixtures, powders, and tablets; he was also allegedly taking steroids. | amphetamine: 1,990 Ur | acute circulatory and respiratory failure in the course of intoxication/1 day |
| 3 | 3-MMC: 3,352 B, 748 VH | F/28 | The woman died in her flat after took about 0.4 g of 3-MMC crystals dissolved in water, had sex with her partner, and then got convulsions, had a nosebleed, and lost consciousness; it was the first time she had taken 3-MMC; previously, she had taken substances of different composition on 3–4 occasions; she was being treated for migraine and was taking hydroxyzine, ketoprofen, and other custom-made medicines. | caffeine: 300 B, 110 VH, codeine: 6 VH | acute circulatory and respiratory failure in the course of intoxication/ 4 days |
| 4 | 3-MMC: 1,710 B | M/52 | The corpse was found without hands and wrists due to activity of animals. | diazepam: 20 B, nordazepam: 6 B | acute circulatory and respiratory failure in the course of intoxication in a person with AIDS and pneumonia/4 days |
| 5 | 4-CMC: 242 B, 720 Ur | M/20 | The man died in an accident—ran in front of a car.  He had been at a party and left it without friends. | alcohol: 2.1 B, 2.9 Ur | polytrauma, especially to the chest/4 days |
| 6 | 4-CMC: 44 B, 662 Ur | M/20 | Road traffic accident. Passenger—sitting in the back seat without wearing a seat belt. | alcohol: 1.5 B, 1.9 Ur | polytrauma/4 days |
| 7 | 4-CMC: 144 B, 5,110 Ur | M/35 | The body was found in a car. The man had a history of heart disease and hypertension. He was taking steroids (stanozolol, clenbuterol, testosterone) and abusing alcohol. | alcohol: 1.4 B, 1.6 Ur | acute circulatory and respiratory failure in the course of intoxication/3 days |
| 8 | 4-CMC: 13 B, 63 Ur | M/38 | The body was found in a flat.  Before going to bed, the man had been drinking beer with a friend; he defecated in bed and a red-brown liquid was coming out of his nose and mouth. He had been having problems but did not reveal their nature; his brother suspects he may have been taking legal highs. |  | acute circulatory and respiratory failure in the course of intoxication/3 days |
| 9 | 4-CMC: 210 B, 257 Ur | F/45 | The body was found in a flat. The woman was suffering from epilepsy, thrombosis, and depression, had suicidal thoughts, cared for her disabled son, and had a drug problem. |  | acute circulatory and respiratory failure in the course of intoxication/6 days |
| 10 | 4-CMC: 223 B, 742 Ur | M/U | The body was found in a flat. A small resealable bag was found in the pocket of the man’s trousers, which contained a white substance. |  | acute circulatory and respiratory failure in the course of intoxication/3 days |
| 11 | 4-CMC: 6 B, 580 Ur | M/41 | Intoxication. The man took drugs at a party; at some point, he suffered a sudden cardiac arrest and died in hospital. |  | acute circulatory and respiratory failure in the course of intoxication/1 days |
| 12 | 4-CMC: 12 B, 54 Ur | M/24 | The body was found in a flat.  The man had been addicted to drugs for 6 years (amphetamine, mephedrone, marijuana), withdrawal signs and symptoms—hallucinations; there were dried plants in the flat. | etizolam: 18 B, 23 Ur | acute circulatory and respiratory failure in the course of intoxication/ 2 days |
| 13 | 4-MEAP: 72 B, 330 Ur | M/31 | The man died in the toilet of the emergency department.  A brown substance was coming out of the man’s mouth. He was homeless and suffered from epilepsy; he admitted to taking ‘white’, which probably meant amphetamine; he also smoked marijuana. | alcohol: 0.5 B, 0.6 Ur, THCCOOH: 17 B, 70 Ur | asphyxia due to aspiration of gastric contents into the respiratory tract in the course of intoxication/3 days |
| 14 | 4-MEAP: 2,740 B, 1,339 Ur | M/48 | The body was found in a flat. | alcohol: 1.7 B, 2.4 Ur | acute circulatory and respiratory failure in the course of intoxication/5 days |
| 15 | 4-MEAP: 10 Ur | M/42 | The body was found in a flat.  The man had complained of feeling unwell for the past week; he suffered from dizziness, showed strange behaviour, refused medical assistance, had a criminal history, abused alcohol and drugs, had hallucinations, and claimed that there were cameras in his house and the family was poisoning him. | amphetamine: 130 B, 1,750 Ur, caffeine: 320 B, 370 Ur | early myocardial infarction in the course of intoxication in a person with pneumonia/5 days |
| 16 | 4-MEAP: 3,121 B | M/35 | The man died in a flat during police intervention;  during the confrontation, he acted irrationally, was strongly agitated, aggressive, sweaty, nervous, did not comply with the officers’ orders, shouted loudly, resisted while being subdued, did not want to leave the flat and get into the police car, and struggled; in front of the Centre for the Prevention of Alcohol-Related Problems, he suddenly started to vomit; he was placed in a lateral position and his handcuffs were removed; the man suffered a sudden cardiac arrest and died.  The man was being treated for drug addiction and suffered from delusions, claiming he ‘would be shot’ and was being ‘pursued’, ‘tracked down’, and ‘followed by a satellite’; he was taking legal highs to be a better person. | amphetamine: 140 B | asphyxia due to aspiration of gastric contents into the respiratory tract in the course of intoxication/2 days |
| 17 | 4-MEAP: 504 B, 860 Ur | M/31 | The body was found in a flat. The man was next to the bed; there was a cigarette holder with an unknown substance in his mouth; he was undergoing psychiatric treatment; from an early age, he suffered from eye and thyroid cancers as well as hepatitis. | citalopram: 50 B, 140 Ur | asphyxia due to aspiration of gastric contents into the respiratory tract in the course of intoxication/1 day |
| 18 | N-ethylpentedrone: 932 B, 140 Ur | M/40 | The body was found in a flat.  The man had been abusing alcohol for 15 years. | alcohol: 3.2 B, 3.5 Ur | acute circulatory and respiratory failure in the course of intoxication/12 days |
| 19 | 4-CEC: 1,633 B, 4,512 Ur | M/21 | The man died during a party in a flat. He was behaving strangely, started to wheeze, got convulsions, his body stiffened, and a white substance came out of his nose; he muttered incomprehensibly and then fell to the floor. He had ingested ‘crystal’ wrapped in toilet paper and took one dose intranasally. He had supposedly been taking drugs on an occasional basis. | alcohol: 1.3 B, 2.3 Ur | early myocardial infarction in the course of intoxication/12 h |
| 20 | 4-CEC: 210 B; 582 Ur | M/29 | Death during physical work. |  | pulmonary haemorrhage in a person with pneumonia and tuberculosis/5 days |
| 21 | NEB: 212 B, 364 Ur | M/27 | The body was found in a flat.  The man had been abusing drugs for a long time. | alcohol: 0.5 B, 0.5 Ur;  caffeine: 13,280 B, 8,980 Ur, lidocaine: 9,670 B, 19,340 Ur, | acute circulatory and respiratory failure in the course of intoxication/3 days |
| 22 | NEB: 53 B, 70 Ur | M/U | Road traffic accident—driver. | alcohol: 1.7 B, 2.4 Ur; amphetamine: 410 Ur; THCCOOH: 38 B, 10 Ur | polytrauma/1 day |
| 23 | N-ethylcathinone: 3,504 B, 4,800 Ur | F/U | Intoxication. The woman took ‘crystal’ with a friend. | amphetamine: 400 B, 1,700 Ur | brain oedema with herniation in the course of intoxication/5 days |
| 24 | α-PVT: 410 B, 603 Ur, 129 ng/g liver, 127 ng/g kidney | M/36 | The body was found in a flat; there were traces of vomit on the couch. |  | acute circulatory and respiratory failure in the course of intoxication/4 days |
| 25 | 3,4-MDPHP: 390 B | M/18 | The body was found in the river. The man had been taking ‘crystal’ and ‘speed’ for two years; he had debts; he stole from his friends; he was afraid for his life and that of his parents. |  | determining the cause of death was difficult due to putrefaction/5 days |
| 26 | α-PVP: 52 B; 1,140 Ur | M/44 | The body was found in a flat. The man abused alcohol. |  | brain oedema with herniation in the course of intoxication/4 days |
| 27 | a-PVP: 62 B | M/24 | The man died in hospital.  The man was running around the premises; he was covered in mud; there were traces of blood on his face and hands; he was very agitated, waved his hands, and shouted that someone was chasing him and that he could see someone; he had dilated pupils and restless eyes; at one point, he ran in front of an oncoming car and continued running; he did not respond to the officers’ orders, put up active resistance, struggled, tried to hit an officer, lost consciousness during the transfer to the police department, and was taken to hospital; he had supposedly been taking drugs, among others mephedrone, and had taken an unspecified substance during a party. |  | acute circulatory and respiratory failure in the course of intoxication/2 days |
| 28 | α-PVP: 20 B, 173 Ur, 29 ng/g kidney, 33 ng/g liver | M/33 | Intoxication. The body remains were found in a burnt car. | HbCO 31%, HCN 7.3% | determining the cause of death was difficult due to charring of the corpse/ 4 days |
| 29 | α-PVP: 410 B, 15,870 Ur | M/U | Intoxication. The man died in hospital~~.~~ after arrival at the ICU. |  | acute circulatory and respiratory failure in the course of intoxication/3 days |
| 30 | α-PVP: 544 B | M/U | The man was found in a flat. | alcohol: 2.0 B  amphetamine: 50 B | acute circulatory and respiratory failure in the course of intoxication/3 days |
| 31 | α-PVP: 182 B, 19 VH | M/28 | The man died during a police intervention.  The man had supposedly been running with his trousers down, accosting passers-by and stopping cars. Upon seeing the officers, he became aggressive and started to flee on foot. During the intervention, the man was subdued by direct coercive measures, i.e. physical force and handcuffs. The man continued to behave aggressively, struggled, shouted words considered offensive and vulgar, and did not follow the instructions given by the offers. Between bouts of aggression, the man admitted to taking psychoactive drugs. |  | acute circulatory and respiratory failure in the course of intoxication/3 days |
| 32 | α-PVP: 20 B | M/27 | The decomposed body was found in a flat.  The gas burners in the flat were turned on; the man was last seen four days earlier; he complained of chest pain and a pricking sensation in the stomach; the flat was a neglected ‘den’; the man supposedly took drugs. | alcohol: 0.6 B | determining the cause of death was difficult due to putrefaction/2 days |
| 33 | N-ethylpentylone: 1,320 B | M/24 | The man died during police intervention in a flat.  He had been found by his female partner in a park under the influence of intoxicants; she took him home, where he behaved strangely, was ‘possessed’, mumbling, aggressive, as if in another world, writhed and threw himself on the ground, punched a hole in the door, tore down the net curtains, was talking to himself, had hallucinations, and saw non-existent people; he supposedly took acid or crystal, the police could not restrain him; the man had a history of alcohol and drug abuse. |  | acute circulatory and respiratory failure in the course of intoxication/ 9 days |
| 34 | Pentedrone: 392 B, 630 Ur | M/U | The man was found in a flat. |  | acute circulatory and respiratory failure in the course of intoxication/3 days |
| 35 | Pentedrone: 817 B, 30,500 Ur | M/26 | The body was found in a stream, partially submerged in water—the man went missing five days earlier. He suffered from peptic ulcer disease; he abused alcohol and took drugs, including 3 g of crystal per week. |  | early myocardial infarction in the course of intoxication/ 4 days |
| 36 | 3,4-DMMC: 5,100 B | M/44 | The body was found in a flat. Near the body there were syringes with needles and in a pocket of the man’s trousers there were three small bags with a white powder in packages labelled ‘Product Imitation: Red Dirt’. The man ran his own business, was known to live on his own, did not drink alcohol, did not use drugs, and for the past six months had been complaining of cardiac pain. |  | acute circulatory and respiratory failure in the course of intoxication/3 days |
| 37 | 4-MMC: 8,404 B, 15,903 Ur | M/24 | The man was found by a neighbour on a staircase. He was being treated for mental problems. |  | early myocardial infarction in the course of intoxication/3 days |
| 38 | PV8: 70 B, 130 Ur | F/37 | Intoxication. Death in hospital. The woman abused alcohol and drugs. | paracetamol 1,490 B, 44,380 Ur, 4‐methylaminoantipyrine 320 B, 12,840 Ur, lidocaine 680 B, 4,630 Ur, dextromethorphan 280 B, 1,980 Ur, drotaverine 250 B, 140 Ur | acute circulatory and respiratory failure in the course of intoxication/2 days |
| 39 | PV8: 260 B, 110 Ur | M/36 | The body was found in a flat. The man was drinking alcohol with his friends at night. He was taking an antibiotic. | clindamycin 1,950 B, 2,930 Ur, alcohol: 1.8 B, 2.4 Ur | acute circulatory and respiratory failure in the course of intoxication/3 days |

U – unknown, M – men, F – female, B – blood, Ur – urine, VH – vitreous humour, PMI – post-mortem interval
